# Pollinator sex matters in competition and coexistence of co-flowering plants

**DOI:** 10.1038/s41598-023-31671-z

**Published:** 2023-03-18

**Authors:** Takefumi Nakazawa, Shigeki Kishi

**Affiliations:** 1 Department of Life Sciences, National Cheng Kung University, Tainan City, Taiwan; 2 Research Center for Agricultural Information Technology, National Agriculture and Food Research Organization, Tsukuba, Japan

**Keywords:** Community ecology, Ecological modelling, Population dynamics, Theoretical ecology

## Abstract

Male and female pollinators often exhibit sex-specific preferences for visiting different flowers. Recent studies have shown that these preferences play an important role in shaping the network structure of pollination mutualism, but little is known about how they can mediate plant-plant interactions and coexistence of competing plants. The ecological consequences of sex-specific pollination can be complex. Suppose that a plant is favoured by female pollinators. They produce male pollinators, who may prefer visiting other competing plants and intensify the negative effects of inter-plant competition. Here, we analysed a simple two plant-one pollinator model with the sex structure of the pollinator. We observed that (i) sex-specific pollination can have complex consequences for inter-plant competition and coexistence (e.g. the occurrence of non-trivial alternative stable states in which one plant excludes or coexists with the other depending on the initial conditions), (ii) male and female pollinators have distinct ecological consequences because female pollinators have a demographic impact owing to reproduction, and (iii) plants are likely to coexist when male and female pollinators prefer different plants. These results suggest that sex-specific pollination is crucial for competition and coexistence of co-flowering plants. Future, pollination research should more explicitly consider the sex-specific behaviour of pollinating animals.

## Introduction

Intraspecific variations, particularly sex differences, are common in sexually reproducing animals. Males and females within the same species often exhibit differences with respect to various biological aspects related to behaviour, ecological niche, life history, morphology, and physiology^1–5^. These sex-specific biological properties can critically affect population dynamics and community structure through the alteration of intraspecific (i.e. mating) and interspecific interactions. For example, males may affect the reproductive success of female partners via Allee effects or sexual harassment, thereby affecting population dynamics^6–8^, whereas sex-specific predation drives complex food-web dynamics^9,10^. However, little is known about the influence of sex-specific mutualism on population dynamics and community structure.

Here, we address how the sex-specific visiting preferences of pollinators affect the competition and coexistence of co-flowering plants. Theoretical studies of plant-pollinator interactions have typically considered the adaptive behaviours of pollinators, ignoring sex-specific visiting preferences^11–13^. Meanwhile, recent empirical studies have reported that the males and females in numerous species exhibit sex-specific preferences for visiting different flowers (or flowers attract different sexes of pollinating animals)^14–16^ (also see Discussion for other examples), which can play an important role in shaping the network structure of pollination mutualism^17–21^. Nevertheless, how sex-specific pollination can mediate plant-plant interactions and their population dynamics remains unclear.

We expect that sex-specific pollination can generate complex ecological consequences. For example, suppose that one plant is favoured by female pollinators. The plant-female interaction contributes to the production of both male and female pollinators. If male pollinators prefer other competing plants, the plant-female interaction may indirectly intensify the negative effects of inter-plant competition, although it directly benefits the plant. Owing to such complexities, theoretical approaches would prove helpful in assessing the ecological consequences of sex-specific pollination.

**Figure 1:**
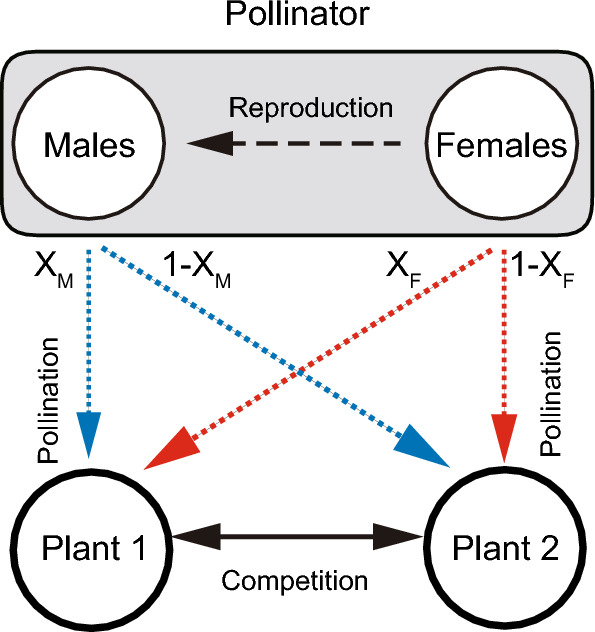
Conceptual illustration of the model analysed in this study. The two competing plants are pollinated by one pollinator species in which male and female pollinators have sex-specific visiting preferences on the two plants. Only female pollinators reproduce offspring. X_M_ and X_F_ are sex-specific visitation frequencies on plant 1.

In this study, we aim to analyse a simple population dynamics model to describe inter-plant competition and coexistence mediated by sex-specific pollination. As an initial study, we adopt the community module approach^22,23^. Specifically, we consider a two plant-one pollinator model with the pollinator’s sex structure (Fig. 1). The three-species system is much simpler than real ecological communities in which numerous plants and animals form a complex network structure. However, the community module approach has the significant advantage that it can identify general processes and qualitative features of ecological communities by focusing on a minimum subset of interacting populations, which provides a mechanistic basis that is useful for studying more complex models in future^22,23^. We also develop the asexual model in which male and female population of the pollinator species are unified into a single population. By comparing the sexual and asexual model, we illustrate that pollinator sex matters in competition and coexistence of co-flowering plants.

## Model

We developed a two plant-one pollinator model with the pollinator’s sex structure as follows:

1a $$\frac{{\mathrm{dP}}_{1}}{\mathrm{dt}}=\left[\left({\mathrm{r}}_{1}+\frac{{\mathrm{a}}_{\mathrm{M},1}\mathrm{M}}{1+{\mathrm{h}}_{\mathrm{M},1}{\mathrm{a}}_{\mathrm{M},1}{\mathrm{P}}_{1}+{\mathrm{h}}_{\mathrm{M},2}{\mathrm{a}}_{\mathrm{M},2}{\mathrm{P}}_{2}}+\frac{{\mathrm{a}}_{\mathrm{F},1}\mathrm{F}}{1+{\mathrm{h}}_{\mathrm{F},1}{\mathrm{a}}_{\mathrm{F},1}{\mathrm{P}}_{1}+{\mathrm{h}}_{\mathrm{F},2}{\mathrm{a}}_{\mathrm{F},2}{\mathrm{P}}_{2}}\right)-{\mathrm{d}}_{\mathrm{1,1}}{\mathrm{P}}_{1}-{\mathrm{d}}_{\mathrm{2,1}}{\mathrm{P}}_{2}\right]{\mathrm{P}}_{1}$$

1b $$\frac{{\mathrm{dP}}_{2}}{\mathrm{dt}}=\left[\left({\mathrm{r}}_{2}+\frac{{\mathrm{a}}_{\mathrm{M},2}\mathrm{M}}{1+{\mathrm{h}}_{\mathrm{M},1}{\mathrm{a}}_{\mathrm{M},1}{\mathrm{P}}_{1}+{\mathrm{h}}_{\mathrm{M},2}{\mathrm{a}}_{\mathrm{M},2}{\mathrm{P}}_{2}}+\frac{{\mathrm{a}}_{\mathrm{F},2}\mathrm{F}}{1+{\mathrm{h}}_{\mathrm{F},1}{\mathrm{a}}_{\mathrm{F},1}{\mathrm{P}}_{1}+{\mathrm{h}}_{\mathrm{F},2}{\mathrm{a}}_{\mathrm{F},2}{\mathrm{P}}_{2}}\right)-{\mathrm{d}}_{\mathrm{1,2}}{\mathrm{P}}_{1}-{\mathrm{d}}_{\mathrm{2,2}}{\mathrm{P}}_{2}\right]{\mathrm{P}}_{2}$$

1c $$\frac{\mathrm{dM}}{\mathrm{dt}}=\mathrm{s}\left({\mathrm{r}}_{\mathrm{P}}+\frac{{\mathrm{b}}_{\mathrm{F},1}{\mathrm{a}}_{\mathrm{F},1}{\mathrm{P}}_{1}+{\mathrm{b}}_{\mathrm{F},2}{\mathrm{a}}_{\mathrm{F},2}{\mathrm{P}}_{2}}{1+{\mathrm{h}}_{\mathrm{F},1}{\mathrm{a}}_{\mathrm{F},1}{\mathrm{P}}_{1}+{\mathrm{h}}_{\mathrm{F},2}{\mathrm{a}}_{\mathrm{F},2}{\mathrm{P}}_{2}}\right)\mathrm{F}-\left({\mathrm{d}}_{\mathrm{M},\mathrm{M}}\mathrm{M}+{\mathrm{d}}_{\mathrm{F},\mathrm{M}}\mathrm{F}\right)\mathrm{M}$$

1d $$\frac{\mathrm{dF}}{\mathrm{dt}}=\left(1-\mathrm{s}\right)\left({\mathrm{r}}_{\mathrm{P}}+\frac{{\mathrm{b}}_{\mathrm{F},1}{\mathrm{a}}_{\mathrm{F},1}{\mathrm{P}}_{1}+{\mathrm{b}}_{\mathrm{F},2}{\mathrm{a}}_{\mathrm{F},2}{\mathrm{P}}_{2}}{1+{\mathrm{h}}_{\mathrm{F},1}{\mathrm{a}}_{\mathrm{F},1}{\mathrm{P}}_{1}+{\mathrm{h}}_{\mathrm{F},2}{\mathrm{a}}_{\mathrm{F},2}{\mathrm{P}}_{2}}\right)\mathrm{F}-\left({\mathrm{d}}_{\mathrm{M},\mathrm{F}}\mathrm{M}+{\mathrm{d}}_{\mathrm{F},\mathrm{F}}\mathrm{F}\right)\mathrm{F}$$

where Pi (i = 1 or 2) is the population abundance of plant species i, and M and F are abundances of the male and female pollinators, respectively. The intrinsic growth rate, r_i_, of the plants is improved by male and female pollinators. In the absence of pollinators, the plants compete with each other (e.g. for nutrients, water, and habitats) via the density-dependent death rate, d_j,i_, which follows the Lotka-Volterra competition model. High values of d_j,i_ indicate that species j has a strong negative effect on species i. The intrinsic growth rate, r_P_, of the pollinator is improved by female pollination. The pollinator has sex-specific visiting rates a_k,1_ and a_k,2_ (k = M or F) on plants 1 and 2, respectively. We assume that the visiting frequency of one plant decreases that of the other plant as a result of allocating limited visiting efforts:

2a $$ {\text{a}}_{{{\text{k}},{1}}} = \alpha_{{\text{k}}} {\text{X}}_{{\text{k}}} $$

2b $$ {\text{a}}_{{{\text{k}},{2}}} = \alpha_{{\text{k}}} \left( {{1} - {\text{X}}_{{\text{k}}} } \right) $$

where α_k_ is the total visiting effort and X_k_ is the visiting preference for plant 1 of each sex. Furthermore, the pollinator has the handling time h_k,i_, conversion efficiency b_F,i_, and density-dependent death rate d_ℓ,k_ (k, ℓ = M or F). The density-dependent death rates of the pollinator imply that male and female pollinators compete with one another for floral resources. Females reproduce offspring with a male ratio of s:1-s. To focus on the effects of sex-specific pollination, we do not consider other factors that could have confounding effects, such as Allee effects^6,7^ and adaptive behaviour^11–13^ in the pollinator. Note also that while the model focuses on the three species (i.e. two flowering plants and one pollinating animal), it is possible to interpret that there are other species in the system. We assume r_i_ > 0, which means that plant populations can grow without pollinators not only due to self-reproduction (including vegetative growth) but also due to pollination services provided by other pollinators not explicitly considered in the model. Likewise, we assume r_P_ > 0, which means that the population growth of the pollinator is partly dependent on other floral resources.

We numerically analysed the model (see Supplementary Information A for analytical approaches using zero-net-growth isoclines). The key parameter was the sex-specific visiting preference X_k_, which was varied between zero and one to assess the effects on inter-plant competition and coexistence. The model predicts four competition outcomes in the absence of pollinators according to the Lotka-Volterra competition model: (i) the two plants coexist, (ii) plant 1 excludes plant 2, (iii) plant 2 excludes plant 1, and (iv) either plant survives depending on the initial conditions. Therefore, we manipulated the density-dependent death rate, d_j,i_, of the plant to represent the four scenarios separately. Furthermore, considering the potential for the occurrence of alternative stable states (ASS), we simulated the model twice for a given parameter set with different initial abundances, where one plant is dominant at the carrying capacity and the other plant is rare; i.e. P_1_(0) = r_1_/d_1,1_ and P_2_(0) = 10^–6^ and vice versa. Initial abundances of the male and female pollinators were M(0) = F(0) = 1. The extinction threshold was set at 10^–6^. Default parameters are r_i_ = 1, d_i,i_ = 0.01, α_k_ = 1, b_k,i_ = 0.1, h_k,i_ = 0.01, d_l,k_ = 0.05, r_P_ = 0.1, and s = 0.5. We then evaluated the steady-state of the population dynamics at t = 10^4^. To obtain robust predictions, we performed sensitivity analysis by varying one parameter while fixing the others to the default values.

For comparison, we also consider the asexual model in which male and female populations of the pollinator species are unified into a single population without sex differences in any aspects. The asexual model is formulated as follows:

3a $$\frac{{\mathrm{dP}}_{1}}{\mathrm{dt}}=\left[\left({\mathrm{r}}_{1}+\frac{{\mathrm{a}}_{1}\mathrm{N}}{1+{\mathrm{h}}_{1}{\mathrm{a}}_{1}{\mathrm{P}}_{1}+{\mathrm{h}}_{2}{\mathrm{a}}_{2}{\mathrm{P}}_{2}}\right)-{\mathrm{d}}_{\mathrm{1,1}}{\mathrm{P}}_{1}-{\mathrm{d}}_{\mathrm{2,1}}{\mathrm{P}}_{2}\right]{\mathrm{P}}_{1}$$

3b $$\frac{{\mathrm{dP}}_{2}}{\mathrm{dt}}=\left[\left({\mathrm{r}}_{2}+\frac{{\mathrm{a}}_{2}\mathrm{N}}{1+{\mathrm{h}}_{1}{\mathrm{a}}_{1}{\mathrm{P}}_{1}+{\mathrm{h}}_{2}{\mathrm{a}}_{2}{\mathrm{P}}_{2}}\right)-{\mathrm{d}}_{\mathrm{1,2}}{\mathrm{P}}_{1}-{\mathrm{d}}_{\mathrm{2,2}}{\mathrm{P}}_{2}\right]{\mathrm{P}}_{2}$$

3c $$\frac{\mathrm{dN}}{\mathrm{dt}}=\left({\mathrm{r}}_{\mathrm{P}}+\frac{{\mathrm{b}}_{1}{\mathrm{a}}_{1}{\mathrm{P}}_{1}+{\mathrm{b}}_{2}{\mathrm{a}}_{2}{\mathrm{P}}_{2}}{1+{\mathrm{h}}_{1}{\mathrm{a}}_{1}{\mathrm{P}}_{1}+{\mathrm{h}}_{2}{\mathrm{a}}_{2}{\mathrm{P}}_{2}}-{\mathrm{d}}_{\mathrm{N}}\mathrm{N}\right)\mathrm{N}$$

where N is the population abundance of the asexual pollinator (see above for parameter definitions). Similarly to the sexual model developed above, we assume that the visiting frequency of one plant decreases that of the other plant

4a $$ {\text{a}}_{{1}} = \alpha {\text{X}} $$

4b $$ {\text{a}}_{{2}} = \alpha \left( {{1} - {\text{X}}} \right) $$

where α is the total visiting effort and X is the visiting preference for plant 1. While manipulating the parameter X, we numerically analysed the asexual model in the same way as the sexual model.

## Results

### Sexual model

**Figure 2:**
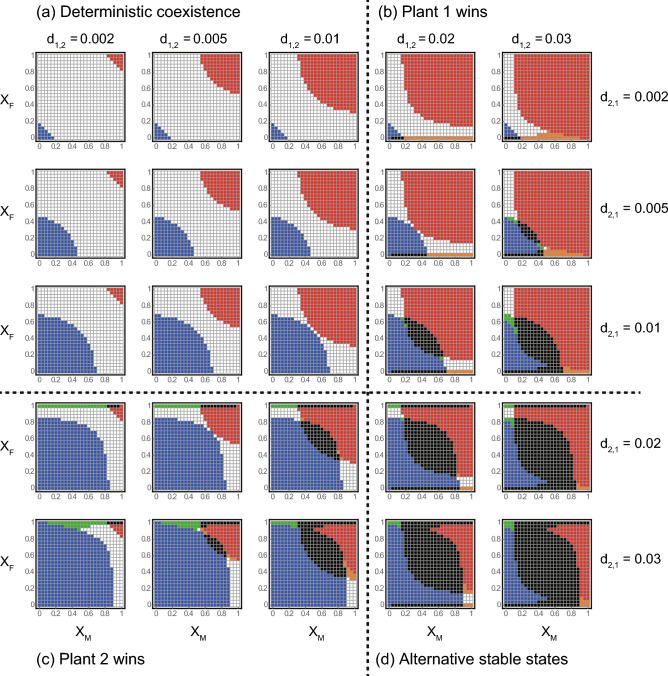
Effects of sex-specific visiting preferences on inter-plant competition and coexistence for different rates of density-dependent plant death. The x and y axes in each panel represent male and female visiting preference on plant 1, respectively. Different colours indicate different steady states: white; the two plants always coexist, red; plant 1 excludes plant 2, blue; plant 2 excludes plant 1, black; either plant survives depending on the initial conditions, orange; plant 1 excludes or coexists with plant 2 depending on the initial conditions, and green; plant 2 excludes or coexists with plant 1 depending on the initial conditions. Panels are arranged according to their density-dependent death rates. From left to light, d_1,2_ = 0.002, 0.005, 0.01, 0.02, and 0.03. From top to bottom, d_2,1_ = 0.002, 0.005, 0.01, 0.02, and 0.03. Note that d_j,i_ indicates the competition effect from the species j to i. Therefore, without pollinators, the plants exhibit (**a**) deterministic coexistence, (**b**) exclusion of plant 2, (**c**) exclusion of plant 1, and (**d**) trivial alternative stable states in which either plant survive depending on the initial conditions.

We separately assessed the ecological consequences of sex-specific pollination for each of the four competition scenarios (see Model). First scenario was where the two plants coexist in the absence of pollinators (Fig. 2a). In this scenario, plant 1 (or 2) outcompetes the other plant when both sexes prefer plant 1 (or 2) (red or blue regions in Fig. 2a). This is because the mutualistic benefits provided by pollinators help the more favoured plant to outcompete the other. Meanwhile, the two plants coexist when neither sex has a strong preference (i.e. X_i_ is close to 0.5) or when they prefer different plants (i.e. males and females prefer plants 1 and 2, respectively, and vice versa) (white regions in Fig. 2a). The parameter space for coexistence in white regions shrinks (or the parameter space for competitive exclusion in red or blue regions expands) with d_1,2_ or d_2,1_ because one plant becomes more competitive.

In the second scenario, plant 1 excludes plant 2 without pollinators (Fig. 2b). Here, plant 1 remains the winner when both sexes prefer it (red regions in Fig. 2b). Otherwise, competition outcomes are complicated. Specifically, two types of ASS occur when females have a strong preference for the less competitive plant 2. In the first type, either plant survives depending on the initial conditions (black regions in Fig. 2b), which is hereafter termed trivial ASS. In the second type, plant 1 outcompetes or coexists with plant 2 depending on the initial conditions (orange regions in Fig. 2b), which is hereafter termed non-trivial ASS. Both types of ASS highlight the enhanced persistence of plant 1 (i.e. blue regions become black and white regions become orange in Fig. 2b). The parameter space expands with d_1,2_, suggesting that the complex dynamics stem from a positive feedback caused by inter-plant competition and female preference, i.e. an increase (or decrease) in the more competitive plant 1 negatively (or positively) affects the less competitive (but more attractive) plant 2, which in turn suppresses (or facilitates) female reproduction and weakens (or strengthens) the mutualism between plant 2 and pollinators. Consequently, plant 1 further increases (or decreases) its population abundance. A similar type of non-trivial ASS also occurs when males have a strong preference for the less competitive plant 2 (i.e. X_M_ is low), females do not have a strong preference (i.e. X_F_ is close to 0.5), and plant 2 has a moderately high value of d_2,1_, where the more favoured plant 2 outcompetes or coexists with the more competitive plant 1 depending on the initial conditions (green regions in Fig. 2b). This type of ASS highlights the enhanced persistence of the less competitive plant 2 (i.e. white regions become green in Fig. 2b). In this parameter space, neither plant can gain substantial benefits from either direct competition or female preference. Therefore, male preferences may cause a positive feedback, i.e. an increase (or a decrease) in plant 2 facilitates (or suppresses) female reproduction and male abundance, which strengthens (or weakens) the mutualism between plant 2 and pollinator via male preference. Consequently, plant 2 further increases (or decreases) its population abundance. The above effects of sex-specific pollination were symmetrically the same for the third scenario, where plant 2 always outcompeted plant 1 without pollinators (Fig. 2c).

In the fourth scenario, either plant survives depending on the initial conditions in the absence of pollinators (Fig. 2d). Here, plant 1 (or 2) tends to be a winner when both sexes strongly prefer plant 1 (or 2) (red or blue regions in Fig. 2d) because of the mutualistic benefits provided by the pollinator. The trivial ASS occur when neither sex had a strong preference (i.e. X_i_ is close to 0.5) or when females strongly preferred either plant (black regions in Fig. 2d). The latter condition implies that females are key in determining the competition outcome, probably because they determine population growth, i.e. a strong association with females leads to positive feedback between plant and pollinator abundance. This mechanism generates non-trivial ASS when the two sexes have strong preferences for different plants (e.g. X_M_ is close to 1 and X_F_ is close to 0, and vice versa), where the plant favoured by females becomes extinct or coexists with the other plant depending on the initial conditions because of the positive feedback (orange and green regions in Fig. 2d).

While the above results show that the ecological consequences of sex-specific pollination can be complicated, it should also be noted that a simple and general pattern emerges across the four scenarios, in that the more favoured plant tends to be a winner, i.e. plant 1 (or 2) outcompetes the other plants when both X_M_ and X_F_ are high (or low). Although this result is intuitive, it conversely indicates that the two plants would coexist (including the occurrence of non-trivial ASS) when male and female pollinators prefer different plants (e.g. X_M_ is close to 1 and X_F_ is close to 0, and vice versa; white, orange, and green regions in Fig. 2). This common pattern clearly illustrates the fundamental importance of the pollinator’s sex-specific visiting preference in the coexistence of competing co-flowering plants. Zero-net-growth isocline analysis confirmed that sex-specific pollination has the varied consequences for inter-plant competition and coexistence as shown by numerical simulations (Supplementary Information A). Moreover, sensitivity analysis showed that these results were generally robust to changes in the parameter values (Supplementary Information B).

### Asexual model

**Figure 3:**
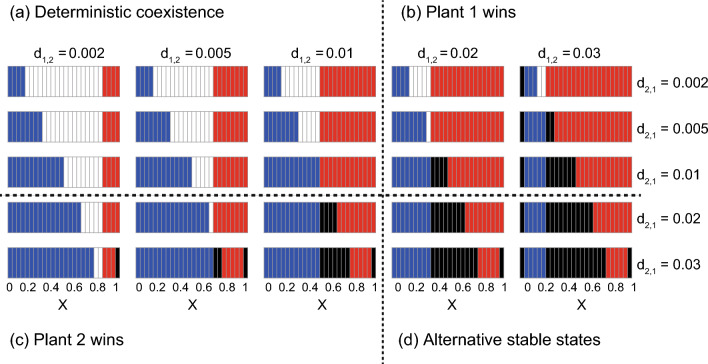
Results of the asexual model showing the effects of visiting preference on inter-plant competition and coexistence for different rates of density-dependent plant death. The horizontal axis in each panel represents the visiting preference on plant 1. The notations are the same as those in Fig. 2.

The asexual model predicted that non-trivial ASS are unlikely to occur (Fig. 3). This is because the complex positive feedbacks (see above) would not work when male and female pollinators are unified into a single population, which also clearly illustrates the importance of sex in the population dynamics of plant-pollinator mutualism. Except for this result, the effects of visiting preference are generally similar to those in the sexual model. That is, the plant more favoured by the pollinator tends to exclude the less favoured plant whereas deterministic coexistence (or trivial ASS) tends to occur when intra-specific competition is stronger (or weaker) than inter-specific competition (see the results along the diagonal line X_M_ = X_F_ in Fig. 2).

## Discussion

We analysed a sex-structured plant-pollinator population dynamics model for the first time. Our results are threefold. First, sex-specific pollination can have complex consequences for inter-plant competition and coexistence because it generates multiple pathways for plant-pollinator interactions (i.e. male and female associations). This is represented by the occurrence of non-trivial ASS, in which one plant outcompetes or coexists with the other plant depending on the initial conditions. Notably, the non-trivial ASS does not occur in the Lotka-Volterra competition system and appears to be caused by the interplay between inter-plant competition and sex-specific pollinator preference. Second, male and female pollinators have distinct ecological consequences for inter-plant competition. In particular, female pollination is more crucial owing to their contribution in reproduction that has demographic impacts. Male pollination can also have dominant effects on inter-plant competition, but it was limited to specific circumstances where female pollinators do not have a strong preference and the two plants have comparatively highly competitive abilities. Third, while the ecological consequences of sex-specific pollination can be complicated, a simple and general pattern emerges, showing that both plants can coexist (including non-trivial ASS) when male and female pollinators prefer different plants. This is analogous to the intuitive result that if the two sexes prefer the same plant, then the more favoured plant can outcompete the other. We emphasise that all these results illustrate the importance of accounting for the sex specificity of pollination behaviour to better understand the competition and coexistence of co-flowering plants.

The complex community consequences of sex-specific pollination (in particular, the occurrence of non-trivial ASS) have practical implications because the parameter conditions are common in nature. We observed that sex-specific pollination will generate non-trivial ASS when direct inter-plant competition is strong and the pollinator (in particular, female pollinators) prefers less competitive plants (Fig. 2b,c), i.e. a trade-off exists between direct inter-plant competition (e.g. for space, nutrients, light, and non-focal pollinators) and pollinator attraction. This trade-off may occur via two mechanisms. One is that the plant more favoured by pollinators may have a lower ability of self-reproduction^24^, and the other is that the plant more favoured by focal pollinators may have a lower ability to attract other pollinators^25^. An intriguing question is whether less competitive plants attract female pollinators to increase long-term fitness through the demographic feedback caused by their reproduction. If this is the case, our model predicts that plant community dynamics are more complex than we have thought.

We showed that, while sex-specific pollination can have complex consequences for the dynamics of inter-plant competition, plant coexistence is more likely to occur when males and females have sex-specific preferences to visit different plants. An increasing number of recent studies have reported that males and females of many pollinator species from various taxa prefer different plants^14–21^. The sex specificity of pollination behaviour originates primarily from sex differences in mating strategy and its nutritional requirements in pollinators. However, with respect to inter-plant competition, the sex specificity of pollination behaviour can be regarded as resource (i.e. pollinator) partitioning that allows the coexistence of competing species^26^. Therefore, we consider that the sex specificity of pollination behaviour, if common in nature, could be regarded as a novel mechanism underlying the diversity of flowering plants. Further model analysis and extension are required to better understand this issue (see below). At the same time, it would also be intriguing to investigate whether and to what degree plants have obtained floral traits to attract a specific sex of pollinators for resource partitioning by evolution or ecological niche shifts.

Male and female pollinators differ not only in visiting preference, as assumed in our model, but also in several other behavioural aspects, such as pollination efficiency and per-visit handling time (i.e. the parameters α_k_ and h_k,i_, respectively, in the model). In bees, females are generally more effective pollinators than conspecific males^27^. If female pollinators have a higher pollination efficiency α_k_ or a lower handling time h_F,i_, our model predicts that the parameter space for non-trivial ASS shrinks and the two plants become more likely to coexist stably, although the general pattern remains largely unchanged (Supplementary Information B). Furthermore, in extreme cases, only one sex may contribute to pollination. For example, *Ficus* plants are pollinated only by female wasps because they provide oviposition sites^28^. In contrast, some species of Orchidaceae and Iridaceae are pollinated only by males as a result of its olfactory mimicry of female sexual pheromones to attract male pollinators^29^. Our model predicted that the plant more favoured by female pollinators has a higher chance of surviving and outcompeting other plants in the case of female-specialised pollination (α_M_ = 0); however, the visiting preference of male pollinators does not affect the outcomes of inter-plant competition, even in the case of male-specialised pollination (α_F_ = 0) (Supplementary Information C). This contrast could also be due to demographic feedback caused by female reproduction, and illustrates the importance of female pollination in regulating inter-plant competition.

The present model is minimal and, thus, can be extended in numerous directions. For example, pollinators may have Allee effects (i.e. a reduction of population growth due to skewed male ratios)^6,7^. If male (or female) pollinators have a much higher (lower) death rate than the other sex, the per-individual fecundity of female pollinators can be diminished due to male limitation, which might have similar effects as decreasing the conversion efficiency b_F,if_. If the pollinator exhibits adaptive behaviour (e.g. switching pollination) to visit major plants^11–13^, it would increase the potential for the occurrence of ASS because the initially dominant plant can have an advantage in attracting pollinators. Moreover, inter-plant competition may occur not only through density-dependent processes but also through frequency-dependent processes, such as reproductive interference, negative effects of interspecific sexual interactions caused by pollen transfer, pollen–pistil interactions, or hybridisation between closely related plant species^30,31^. Reproductive interference causes ASS in competition dynamics due to positive feedback, in which the initially dominant species can further suppress other species^32^. We infer that if sex-specific pollination and reproductive interference work together, ASS would be more likely to occur, thereby preventing the coexistence of co-flowering plants.

**Figure 4:**
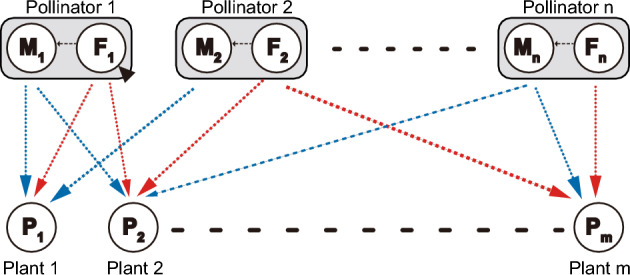
Conceptual illustration of a bipartite network model with m plant species and n pollinator species.

Another promising direction for future research is to extend the present module model into bipartite networks in which population dynamics of multiple plants and pollinators are explicitly described (Fig. 4). One possible model is as follows:

5a $$\frac{{\mathrm{dP}}_{\mathrm{m}}}{\mathrm{dt}}=\left[\left({\mathrm{r}}_{\mathrm{m}}+\sum_{{\mathrm{n}}^{{^{\prime}}}=1}^{\mathrm{n}}\frac{{\mathrm{a}}_{\mathrm{M},\mathrm{m},{\mathrm{n}}^{{^{\prime}}}}{\mathrm{M}}_{{\mathrm{n}}^{{^{\prime}}}}}{1+\sum_{{\mathrm{m}}^{{^{\prime}}}=1}^{\mathrm{m}}{\mathrm{h}}_{\mathrm{M},{\mathrm{m}}^{{^{\prime}}},{\mathrm{n}}^{{^{\prime}}}}{\mathrm{a}}_{\mathrm{M},{\mathrm{m}}^{{^{\prime}}},{\mathrm{n}}^{{^{\prime}}}}{\mathrm{P}}_{{\mathrm{m}}^{{^{\prime}}}}}+\sum_{{\mathrm{n}}^{{^{\prime}}}=1}^{\mathrm{n}}\frac{{\mathrm{a}}_{\mathrm{F},\mathrm{m},\mathrm{n{^{\prime}}}}{\mathrm{F}}_{\mathrm{n{^{\prime}}}}}{1+\sum_{{\mathrm{m}}^{{^{\prime}}}=1}^{\mathrm{m}}{\mathrm{h}}_{\mathrm{F},\mathrm{m{^{\prime}}},\mathrm{n{^{\prime}}}}{\mathrm{a}}_{\mathrm{F},\mathrm{m{^{\prime}}},\mathrm{n{^{\prime}}}}{\mathrm{P}}_{\mathrm{m{^{\prime}}}}}\right)-\sum_{{\mathrm{m}}^{{^{\prime}}}=1}^{\mathrm{m}}{\mathrm{d}}_{\mathrm{m{^{\prime}}},\mathrm{m}}{\mathrm{P}}_{\mathrm{m{^{\prime}}}}\right]{\mathrm{P}}_{\mathrm{m}}$$

5b $$\frac{{\mathrm{dM}}_{\mathrm{n}}}{\mathrm{dt}}=\frac{1}{2}\left({\mathrm{r}}_{\mathrm{P},\mathrm{n}}+\frac{\sum_{{\mathrm{m}}^{{^{\prime}}}=1}^{\mathrm{m}}{\mathrm{b}}_{\mathrm{F},\mathrm{m{^{\prime}}}}{\mathrm{a}}_{\mathrm{F},\mathrm{m{^{\prime}}}}{\mathrm{P}}_{\mathrm{m{^{\prime}}}}}{1+\sum_{{\mathrm{m}}^{{^{\prime}}}=1}^{\mathrm{m}}{\mathrm{h}}_{\mathrm{F},\mathrm{m{^{\prime}}}}{\mathrm{a}}_{\mathrm{F},\mathrm{m{^{\prime}}}}{\mathrm{P}}_{\mathrm{m{^{\prime}}}}}\right){\mathrm{F}}_{\mathrm{n}}-\sum_{{\mathrm{n}}^{{^{\prime}}}=1}^{\mathrm{n}}\left({\mathrm{d}}_{\mathrm{M},\mathrm{M},\mathrm{n{^{\prime}}}}{\mathrm{M}}_{\mathrm{n{^{\prime}}}}+{\mathrm{d}}_{\mathrm{F},\mathrm{M},\mathrm{n{^{\prime}}}}{\mathrm{F}}_{\mathrm{n{^{\prime}}}}\right){\mathrm{M}}_{\mathrm{n}}$$

5c $$\frac{{\mathrm{dF}}_{\mathrm{n}}}{\mathrm{dt}}=\frac{1}{2}\left({\mathrm{r}}_{\mathrm{P},\mathrm{n}}+\frac{\sum_{{\mathrm{m}}^{{^{\prime}}}=1}^{\mathrm{m}}{\mathrm{b}}_{\mathrm{F},\mathrm{m{^{\prime}}}}{\mathrm{a}}_{\mathrm{F},\mathrm{m{^{\prime}}}}{\mathrm{P}}_{\mathrm{m{^{\prime}}}}}{1+\sum_{{\mathrm{m}}^{{^{\prime}}}=1}^{\mathrm{m}}{\mathrm{h}}_{\mathrm{F},\mathrm{m{^{\prime}}}}{\mathrm{a}}_{\mathrm{F},\mathrm{m{^{\prime}}}}{\mathrm{P}}_{\mathrm{m{^{\prime}}}}}\right){\mathrm{F}}_{\mathrm{n}}-\sum_{{\mathrm{n}}^{{^{\prime}}}=1}^{\mathrm{n}}\left({\mathrm{d}}_{\mathrm{M},\mathrm{F},\mathrm{n{^{\prime}}}}{\mathrm{M}}_{\mathrm{n{^{\prime}}}}+{\mathrm{d}}_{\mathrm{F},\mathrm{F},\mathrm{n{^{\prime}}}}{\mathrm{F}}_{\mathrm{n{^{\prime}}}}\right){\mathrm{F}}_{\mathrm{n}}$$

where m and n are the number pf plant and pollinator species, respectively. To parameterise the model, we need empirical data on the structural properties of plant–pollinator networks. Studies have shown that female pollinators have a wider floral niche breadth than male pollinators in plant–insect pollinator networks^18,19^. Likewise, Maglianesi et al.^21^ reported that female pollinators are more generalists in hummingbird communities. Therefore, this pattern would be widely observed across plant–pollinator networks. Males and females differ not only in floral niche breadth, but also in the type of flower visited and per-visit pollen transfer efficiency, but the available evidence is still limited^19^. Other empirical questions also remain open, such as the extent to which the sexes differ in total visitation effort (i.e. the parameter α_k_ in the model) and the factors determining sex-specific floral niches (e.g. taxonomic identity, morphology, and local biodiversity). It is important to study such issues, considering the possibility that male and female pollinators have distinct consequences for inter-plant competition and sex differences in pollination behaviour are crucial for plant coexistence.

In conclusion, the present study sheds new light on theoretical approaches to plant community dynamics because, to date, the sex specificity of pollination behaviour has rarely been considered in the context of inter-plant interactions. We specifically focused on pollination mutualism. This idea would be potentially applicable to seed dispersal mutualism as well, because male and female animals often exhibit different nutrient requirements and thus different food preferences^1^. More research efforts should be devoted to a better understanding of the role of sex in plant-animal mutualism and its community consequences.

### Supplementary Information

Supplementary Information 1.

Supplementary Information 2.

## Data Availability

A Mathematica codes for file for simulating population dynamics is available at Supplementary Information.
