# Author Correction: Pollinator sex matters in competition and coexistence of co-flowering plants

**DOI:** 10.1038/s41598-023-46412-5

**Published:** 2023-11-02

**Authors:** Takefumi Nakazawa, Shigeki Kishi

**Affiliations:** 1 Department of Life Sciences, National Cheng Kung University, Tainan City, Taiwan; 2 Research Center for Agricultural Information Technology, National Agriculture and Food Research Organization, Tsukuba, Japan

Correction to: *Scientific Reports* 10.1038/s41598-023-31671-z, published online 18 March 2023

The Funding section in the original version of this Article was incomplete.

“This study was supported by National Science and Technology Council, Taiwan.”

now reads:

“This study was supported by National Science and Technology Council, Taiwan. This research was supported in part by Higher Education Sprout Project, Ministry of Education to the Headquarters of University Advancement at National Cheng Kung University NCKU.”

The original Article has been corrected.

