# Supplementary material for: Pollinator sex matters in competition and coexistence of co-flowering plants
Source: Sci Rep. 2023 Mar 18;13:4497. doi: 10.1038/s41598-023-31671-z (PMC10024751; doi:10.1038/s41598-023-31671-z)
Supplement: Supplementary file 1 — Supplementary Information 1. [file 41598_2023_31671_MOESM1_ESM.docx]

ClearAll["Global`*"]

r1=r2=1;

{d12, d21}={0.01,0.01};

K1=r1/d11; K2=r2/d22;

AF=AM=1;

XM=0.7; XF=0.3;

aF1=XF AF; aF2=(1-XF)AF;

aM1=XM AM; aM2=(1-XM)AM;

bF1=bF2=0.1;

hF1=hF2=hM1=hM2=0.01;

d=0.05;

d11=d22=0.01;

dP1dt=(r1(1+(aF1 F[t])/(1+hF1 aF1 P1[t]+hF2 aF2 P2[t])+(aM1 M[t])/(1+hM1 aM1 P1[t]+hM2 aM2 P2[t]))-d11 P1[t]-d21 P2[t])P1[t];

dP2dt=(r2(1+(aF2 F[t])/(1+hF1 aF1 P1[t]+hF2 aF2 P2[t])+(aM2 M[t])/(1+hM1 aM1 P1[t]+hM2 aM2 P2[t]))-d12 P1[t]-d22 P2[t])P2[t];

dFdt=(bF1 aF1 P1[t]+bF2 aF2 P2[t])/(2(1+hF1 aF1 P1[t]+hF2 aF2 P2[t])) F[t]-d(F[t]+M[t])F[t];

dMdt=(bF1 aF1 P1[t]+bF2 aF2 P2[t])/(2(1+hF1 aF1 P1[t]+hF2 aF2 P2[t])) F[t]-d(F[t]+M[t])M[t];

T=10000;

δ=10^-6;

sol1=NDSolve[{P1'[t]==dP1dt, P2'[t]==dP2dt, F'[t]==dFdt,M'[t]==dMdt,P1[0]==K1,P2[0]==δ,M[0]==1,F[0]==1},{P1,P2,F,M},{t,0,T}];

sol2=NDSolve[{P1'[t]==dP1dt, P2'[t]==dP2dt, F'[t]==dFdt,M'[t]==dMdt,P1[0]==δ,P2[0]==K2,M[0]==1,F[0]==1},{P1,P2,F,M},{t,0,T}];

Plot[{P1[t]/.sol1, P2[t]/.sol1},{t,0,T}, PlotRange->All]
