# Supplementary material for: Pollinator sex matters in competition and coexistence of co-flowering plants
Source: Sci Rep. 2023 Mar 18;13:4497. doi: 10.1038/s41598-023-31671-z (PMC10024751; doi:10.1038/s41598-023-31671-z)
Supplement: Supplementary file 2 — Supplementary Information 2. [file 41598_2023_31671_MOESM2_ESM.docx]

**Supplementary Information A**

Here, we explain how to perform zero-net-growth isocline analysis. First, we assumed that for analytical tractability, male and female pollinators have comparable rates of density-dependent death (i.e. d_ℓ,k_ = d). With this assumption, the following equations hold at the steady state of pollinator population dynamics:

$\frac{1}{2}\left( r_{P}+\frac{b_{F,1}a_{F,1}{P_{1}}^{*}+b_{F,2}a_{F,2}{P_{2}}^{*}}{1+h_{F,1}a_{F,1}{P_{1}}^{*}+h_{F,2}a_{F,2}{P_{2}}^{*}} \right)F^{*}-d\left( M^{*}+F^{*} \right)M^{*}=0$

$\frac{1}{2}\left( r_{P}+\frac{b_{F,1}a_{F,1}{P_{1}}^{*}+b_{F,2}a_{F,2}{P_{2}}^{*}}{1+h_{F,1}a_{F,1}{P_{1}}^{*}+h_{F,2}a_{F,2}{P_{2}}^{*}} \right)-d\left( M^{*}+F^{*} \right)=0$.

Combining the equations, we obtain

$\frac{1}{2}\left( r_{P}+\frac{b_{F,1}a_{F,1}{P_{1}}^{*}+b_{F,2}a_{F,2}{P_{2}}^{*}}{1+h_{F,1}a_{F,1}{P_{1}}^{*}+h_{F,2}a_{F,2}{P_{2}}^{*}} \right)=d\left( M^{*}+F^{*} \right)\frac{M^{*}}{F^{*}}=d\left( M^{*}+F^{*} \right)$,

which leads to M^*^ = F^*^. Furthermore, from the pollinator equilibrium equation, we obtain

$M^{*}=F^{*}=\frac{1}{4d}\left( r_{P}+\frac{b_{F,1}a_{F,1}{P_{1}}^{*}+b_{F,2}a_{F,2}{P_{2}}^{*}}{1+h_{F,1}a_{F,1}{P_{1}}^{*}+h_{F,2}a_{F,2}{P_{2}}^{*}} \right)$.

Substituting this into the following steady-state equation for the two plants yields the zero-net growth isoclines:

$r_{1}+\frac{a_{M,1}M^{*}}{1+h_{M,1}a_{M,1}{P_{1}}^{*}+h_{M,2}a_{M,2}{P_{2}}^{*}}+\frac{a_{F,1}F^{*}}{1+h_{F,1}a_{F,1}{P_{1}}^{*}+h_{F,2}a_{F,2}{P_{2}}^{*}}-d_{1,1}{P_{1}}^{*}-d_{2,1}{P_{2}}^{*}=0$

$r_{2}+\frac{a_{M,2}M^{*}}{1+h_{M,1}a_{M,1}{P_{1}}^{*}+h_{M,2}a_{M,2}{P_{2}}^{*}}+\frac{a_{F,2}F^{*}}{1+h_{F,1}a_{F,1}{P_{1}}^{*}+h_{F,2}a_{F,2}{P_{2}}^{*}}-d_{1,2}{P_{1}}^{*}-d_{2,2}{P_{2}}^{*}=0$

By using the zero-net-growth isolines, we plotted the dynamical trajectory on the two-dimensional plane of P_1_ and P_2_, which confirmed the results of numerical simulations (Fig. S1).

**Figure S1.** Results of zero-net-growth isocline analysis. Red and blue lines indicate the zero-net-growth isoclines for the plant 1 and 2, respectively. Black circles represent the equilibria where the dynamics converge. (a-c) The two plants coexist without pollinators. The density-dependent death rates of the plants are (d_1,2_, d_2,1_) = (0.005, 0.005). Sex-specific visiting preferences are (X_M_, X_F_) = (a) (0.1, 0.1), (b) (0.5, 0.5), and (c) (0.9, 0.9). These parameter combinations correspond to three possible outcomes in figure 2a: (a) plant 2 excludes plant 1 (blue regions), (b) the two plants always coexist (white regions), and (c) plant 1 excludes plant 2 (red regions). (d-i) The plant 1 excludes the plant 2 without pollinators. The density-dependent death rates of the plants are (d_1,2_, d_2,1_) = (0.03, 0.005). Sex-specific visiting preferences are (X_M_, X_F_) = (d) (0.05, 0.05), (e) (0.1, 0.4), (f) (0.25, 0.25), (g) (0.05, 0.5), (h) (0.5, 0.05), and (i) (0.5, 0.5). These parameter combinations correspond to six possible outcomes in figure 2b: (d) plant 2 excludes plant 1 (blue regions), (e) non-trivial alternative stable states occur in which plant 2 excludes or coexists with plant 1 depending on the initial conditions (green regions), (f) trivial alternative stable states occur in which either plant survives depending on the initial conditions (black regions), (g) the two plants always coexist (white regions), (h) non-trivial alternative stable states occur in which plant 1 excludes or coexists with plant 2 depending on the initial conditions, and (i) plant 1 excludes plant 2 (red regions). (j-n) Trivial alternative stable states occur without pollinators. The density-dependent death rates of the plants are (d_1,2_, d_2,1_) = (0.03, 0.03). Sex-specific visiting preferences are (X_M_, X_F_) = (j) (0.1, 0.1), (k) (0.1, 0.9), (ℓ) (0.2, 0.2), (m) (0.9, 0.1), and (n) (0.9, 0.9). These parameter combinations correspond to five possible outcomes in figure 2d: (j) plant 2 excludes plant 1 (blue regions), (k) non-trivial alternative stable states occur in which plant 2 excludes or coexists with plant 1 depending on the initial conditions (green regions), (ℓ) trivial alternative stable states occur in which only either plant survives depending on the initial conditions (black regions), (m) non-trivial alternative stable states occur in which plant 1 excludes or coexists with plant 2 depending on the initial conditions, and (n) plant 1 excludes plant 2 (red regions). Other parameters are the same as those in figure 2.

**Supplementary Information B**

Here, we performed a sensitivity analysis by varying one parameter while fixing the other parameters at default values. We found that the results in figure 2 are generally robust to changes in the parameter values (Fig. S2).

**Figure S2.** Results of sensitivity analysis. For each panel, one parameter was varied: (a) default, (b) r_1_ was varied from 1 to 1.5, (c) α_F_ was varied from 1 to 1.5, (d) b_F_ was varied from 0.1 to 0.15, (e) h_F,1_ was varied from 0.01 to 0.005, (f) d_F,M_ was varied from 0.05 to 0.075, (g) d_M,F_ was varied from 0.05 to 0.025, (h) r_P_ was varied from 0.1 to 0.2, and (i) s was varied from 0.5 to 0.25. For each parameter set, we used four combinations of density-dependent death rates of the plants: (d_1,2_, d_2,1_) = (0.005, 0.005), (0.03, 0.005), (0.005, 0.03), and (0.03, 0.03). These four combinations correspond to the four possible scenarios (i.e. deterministic coexistence, exclusion of plant 2, exclusion of plant 1, and trivial alternative stable states) in the absence of pollinators.

**Supplementary Information C**

Here, we performed an additional sensitivity analysis to explore the effects of sex-specialised pollination. We considered the two extreme cases that only females or males contributed to pollination. The results showed that the visiting preferences of female pollinators can critically affect inter-plant competition in the case of female-specialised pollination. Specifically, plants favoured by female pollinators have a higher chance of surviving and outcompeting the less favoured plant (Fig. S3a). However, the visiting preference of male pollinators did not affect the inter-plant competition, even in the case of male-specialised pollination (Fig. S3b).

**Figure S3.** Results of sex-specialised pollination. (α_M_, α_F_) = (a) (0, 1) and (b) (1, 0). Other notations are the same as those in figure 2.
